# Practices and Trends of Machine Learning Application in Nanotoxicology

**DOI:** 10.3390/nano10010116

**Published:** 2020-01-08

**Authors:** Irini Furxhi, Finbarr Murphy, Martin Mullins, Athanasios Arvanitis, Craig A. Poland

**Affiliations:** 1 Department of Accounting and Finance, Kemmy Business School, University of Limerick, V94PH93 Limerick, Ireland; finbarr.murphy@transgero.eu (F.M.); martin.mullins@transgero.eu (M.M.); 2 Transgero Limited, Newcastle, V42V384 Limerick, Ireland; 3 Department of Mechanical Engineering, Environmental Informatics Research Group, Aristotle University of Thessaloniki, 54124 Thessaloniki Box 483, Greece; at.arvanitis@dei.com.gr; 4 ELEGI/Colt Laboratory, Queen’s Medical Research Institute, 47 Little France Crescent, University of Edinburgh, Edinburgh EH16 4TJ, Scotland, UK; craig.poland@ed.ac.uk

**Keywords:** machine learning, in silico, computational, nanoparticle, nanoforms, nanotoxicology

## Abstract

Machine Learning (ML) techniques have been applied in the field of nanotoxicology with very encouraging results. Adverse effects of nanoforms are affected by multiple features described by theoretical descriptors, nano-specific measured properties, and experimental conditions. ML has been proven very helpful in this field in order to gain an insight into features effecting toxicity, predicting possible adverse effects as part of proactive risk analysis, and informing safe design. At this juncture, it is important to document and categorize the work that has been carried out. This study investigates and bookmarks ML methodologies used to predict nano (eco)-toxicological outcomes in nanotoxicology during the last decade. It provides a review of the sequenced steps involved in implementing an ML model, from data pre-processing, to model implementation, model validation, and applicability domain. The review gathers and presents the step-wise information on techniques and procedures of existing models that can be used readily to assemble new nanotoxicological in silico studies and accelerates the regulation of in silico tools in nanotoxicology. ML applications in nanotoxicology comprise an active and diverse collection of ongoing efforts, although it is still in their early steps toward a scientific accord, subsequent guidelines, and regulation adoption. This study is an important bookend to a decade of ML applications to nanotoxicology and serves as a useful guide to further in silico applications.

## 1. Introduction

Nanomaterials/nanoforms (NMs) display high heterogeneity regarding their physicochemical (p-chem) properties, quantum-mechanical properties, and, as such, their toxicological impact, which renders assessing their risk a case-by-case challenge. Traditional hazard assessment relies mostly on in vivo testing that poses technical challenges, e.g., regarding the validity of extrapolation to humans, ethical dilemmas, but also comes with high resource demands in cost and time [1]. Such an approach is not conducive to efficient identification and mitigation of possible risks, especially within emerging technologies where the pace of development is rapid. There is a momentum from scientific and policy influencing bodies globally to promote in silico models as alternatives methods in compliance with the 3R (Replacement, Reduction, and Refinement) principles for reducing the use of animals in research. Moreover, developing the knowledge base needed for robust modelling for predicting NM properties, exposure, and hazard potential would also improve the design of new materials while maximizing utility and minimizing adverse biological effects (safe-by-design) [2,3]. In order to investigate the potential of modelling the toxicity and properties of NMs, the European Commission has funded several modelling projects [4,5]. However, in silico tools are not yet accepted by regulators as a stand-alone solution due to a lack of standardization, but as a complementary tool [6,7].

Diverse computational models have been developed during the last decade for predicting toxicological properties or the adverse effects of NMs. As the use of computational tools is increasing, the goal of this manuscript is to provide a snapshot of all processing steps in model implementations of the last decade, in order to provide paradigms that can lead to more robust model building. A Quantitative Structure-Activity Relationship (QSAR) and Quantitative Structure-Property Relationship (QSPR) are among the most used tools in the nanotoxicology prediction. Villaverde et al. [8] analyzed QSAR/QSPR tools for risk assessment, modeling methods, and validation procedures with regard to their potential for meeting requirements within the European legislative framework for authorization of nano-formulations. The authors argued that the standardization of protocols is needed, even for high-quality and well-described datasets. Quik et al. [9] analyzed available models and their parametrization related to NM properties for risk assessment. The authors showed an opportunity for development of new predictive in silico methods when full mechanistic functioning of the NM-biological surfaces system is accounted for. The Nanoinformatics Roadmap 2030 [5] is a compilation of state-of-the-art commentaries from multiple scientific fields dealing with issues involving NM risk assessment and governance. The authors addressed three recognized challenges that nanoinformatics face in general such as limited data sets, limited data access, and regulatory requirements for validating and accepting computational models. The authors warned for the need of interconnecting harmonized databases in a framework that entails early use of data for regulatory purposes, e.g., read-across method of filling data gaps, to prevent unstructured progress in generating data.

Schemes for clustering NMs have been proposed and reviewed elsewhere [10,11,12]. Lamon et al. [11] addressed categorization schemes, grouping for read-across approaches and computational applications for ranking NMs. The authors stated that the few studies dealing with NM similarities used non user-friendly tools on limited datasets. The authors suggested that toxicity datasets and nano-specific properties should both be investigated to identify groups of NMs. Giusti et al. [12] noted how in silico methods contribute at different stages of NM grouping such as in developing vs. supporting initial grouping hypotheses. The methods to be used vary, from read-across, unsupervised, and supervised machine learning (ML) methods to several QSAR approaches.

The Organization for Economic Cooperation and Development (OECD) have published a set of validation principles of QSAR models [13]. These principles detect that models should have a well-defined endpoint, unambiguous algorithm, defined domain of applicability, appropriate measure of goodness-of-fit, robustness and predictivity, and mechanistic interpretation. Such principles are fundamental and must be taken into account when dealing with in silico models in general. More in depth information about the OECD model validation principles can be found elsewhere [14,15] including suggestions for extension. Puzyn et al. [4] discussed relevant considerations to be taken into account when evaluating QSAR models, according to the OECD principles, including the quality of the data and the model results reproducibility. Basei et al. [14] critically analyzed existing approaches of ML techniques based on their predictive ability regarding health hazard endpoints and proposed possible developments. The authors provided adopted criteria to evaluate computational tools that predict nanotoxicity, inspired by the OECD principles. Lamon et al. [16] proposed the use of harmonized model reporting templates or QSAR Model Reporting Format (QMRF), for systematically describing models of NM regulatory risk assessments. The templates include an adaptation of the QMRF, a reporting template for Physiologically-Based PharmacoKinetic (PBPK) and environmental exposure models, applicable to NMs. The authors demonstrated the value of these templates on reporting different models and overviewing the landscape of available models for NMs. ToxRTool (Toxicological data Reliability assessment Tool), which is a compilation of reliability assessment questions, can also be employed to asses meta-analyzed studies for human health hazard assessments [17].

Based on the above reviews, it is evident that a lot of effort and research is needed so that in silico tools are both accepted by regulators and implemented in a harmonized way to maximize their utility. The applicability domain was discussed through the existing reviews as well as the limitations of the dataset (e.g., size), the lack of nano-specific descriptors, and the validation performance. This paper provides an extensive up-to-date review focusing on the techniques that are used to predict a human health and/or environmental outcome including selection of algorithms and the employed performance metrics and applicability domain methods. The review gathers and presents step-wisely information on techniques and procedures of existing models that computational toxicologists and researchers can adopt to assemble their own nanotoxicological in silico studies.

Our research finds that data preprocessing, including selecting the features, addressing class imbalance, normalizing data and methodological splitting, is essential before model implementation. Proper model performance metrics and statistics, including uncertainty and sensitivity analysis, are indispensable elements of model evaluation. This study shows that tree algorithms (i.e., random forest) are the most common ML used due to insensitivity of data defects, resistance to overfitting, and robustness in small datasets. Regression models traditionally used in classic QSARs are still common but trending shifts are toward nonlinear algorithms. Artificial Neural Networks have a great deal of potentiality but data paucity limits their use for the time being. This review is preceded by another analysis of the literature identified herein, focusing on data collection, curation, and utilization [18] as a precursor to data pre-processing and model implementation.

## 2. Methods

### 2.1. Search Design

In order to investigate ML models in the field of nanotoxicology, we explored several sources of the peer-reviewed scientific literature and reports executing a systematic Boolean search with key terms, such as “nanoparticle,” “nanomaterial,” “in silico,” “computational,” “machine learning,” “model,” and “nanotoxicity.” These were used to form defined multiple search strings, which were applied to publicly available electronic search engines (*Google Scholar*, *ScienceDirect*, *Web of Science*, and *PubMed*) with the aim of being able to discover studies that implement an ML model to predict nanotoxicity (Table 1). The final technical report of NanoComput project, “Evaluation of the availability and applicability of computational approaches in the safety assessment of nanomaterials” carried out by the European Commission’s Joint Research Centre (JRC) was taken into consideration for studies before 2017 [15].

### 2.2. Eligibility and Exclusion Criteria

We focused on ML models predicting ecotoxicological (e.g., effects on terrestrial organisms, aquatic toxicity, etc.) and human health toxicological endpoints. In this review, the endpoint is a specific biological effect defined in terms of biological target structure and associated changes in tissue structures and/or other parameters [19]. Therefore, studies predicting properties of NPs such as solubility, dispersion, absorption, zeta potential, partition coefficients, Poisson’s ratio or Young’s Modulus, and environmental outcomes (e.g., bioaccumulation, degradation) were not included. In addition, Physiologically-Based PharmacoKinetic (PBPK) modelling was not addressed in this study since it has been addressed recently elsewhere [20,21].

As summarized in Table 1, the literature review utilized different inclusion criteria. Additionally, studies should (i) focus on the model implementation, (ii) have been published during the last decade, (iii) published in English, and (iv) published in peer-reviewed journals or final project reports. The search restrictions were applied to the title, abstract, and keywords. In addition, manual searches were performed addressing reference lists from published papers in order to identify any additional studies overlooked by the electronic search. Using this structured approach, 86 articles implementing ML models for nanotoxicity prediction, published in the last decade, were identified.

### 2.3. Analysis

**Figure 1:**
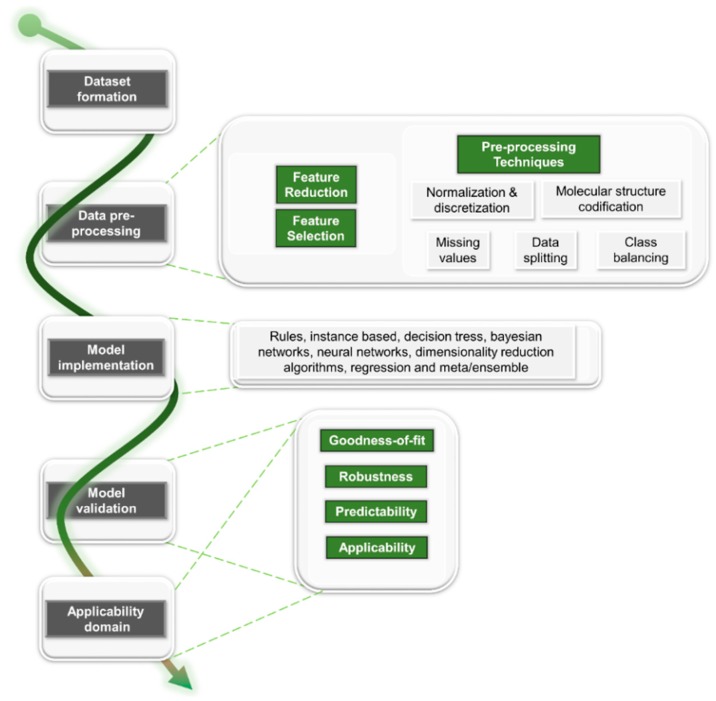
A summarized general roadmap for implementing a model in the field of nanotoxicology. The roadmap can be divided into five main parts: dataset formation, data pre-processing, model implementation, model validation, and applicability domain.

Each of the 86 identified articles were reviewed in detail and information related to the feature selection process, data processing techniques, model implementation (model category and algorithm), model validation, and applicability domain was extracted. There were no definite guidelines in choosing pre-processing techniques, model implementation, and validation metrics in order to assess the performance and applicability domain of computational models. Figure 1 shows a summary diagram of the process steps applied to the identified, which follows a generalized roadmap from data extraction to model validation and applicability domain.

This roadmap comprises five main sequential parts and our focus herein are the four subparts consisting of data pre-processing, model implementation, validation, and applicability domain. The first sequential part of Figure 1 such as dataset formation that addresses the endpoint and an in depth-analysis (mapping) of the most common endpoints predicted in the reviewed studies has been addressed in a separate, companion article. However, it is briefly discussed hereafter [18].

## 3. Results

### 3.1. Dataset Formation

The first part in processes of ML model implementation, Dataset formation (Figure 1), contains four subparts. First, data collection is carried out, either from existing literature and databases or from new data experimentally created. A combination of the previously mentioned sources can also be used. Second, the information on NPs is extracted, including nano-specific descriptors (size, coating, zeta potential, etc.) and the type of NPs (metal, metal oxide, carbon-based, etc.), derived either from the data sources or elsewhere, e.g., the manufacturer data sheets. Besides nano-specific descriptors as inputs and theoretical descriptors can be generated using available software and used as input data. Third, inputs including study design information is attained, such as the testing system (in vitro, in vivo), species (human, bacteria, etc.), tissue (lung, kidney, etc.), exposure conditions (dose, duration), and in vitro experimental features (e.g., cell line: A549, Caco2, etc.) or detailed toxicological assays. Lastly, the toxicological endpoint of the study is obtained to be used as the predicted output of the model. A detailed description of the datasets used can be found elsewhere [18].

### 3.2. Data Pre-Processing

The second part in the processes of ML model implementation, after dataset formation, consists of data-preprocessing methods such as features reduction, features selection, and data pre-processing techniques (Figure 1).

#### 3.2.1. Feature Reduction

After the generation of theoretical descriptors of NMs, an initial reduction can be performed among variables to reduce the amount of irrelevant or redundant information [22]. Such cases include constant or near constant descriptors with low variance, descriptors with missing or zero values, and collinear highly correlated pairs of variables. In the case of correlated variables, the one with higher correlation with the endpoint is chosen in developing the model [23]. In addition to descriptors’ reduction, a feature selection process is followed in order to optimize the performance of the model. Feature selection may be appropriate for two key reasons including to avoid overfitting training data and, second, to enable expert assessment of the mechanistic basis for the model [4,24]. Almost half of the identified studies applied some form of a feature selection process to their initial dataset.

#### 3.2.2. Feature Selection

In building a QSAR model, statistical performance metrics of the best, (one- to five-) variable models selected by feature selection are calculated [25]. As the rule of thumb, model validation is performed by increasing the number of involved variables and assessing performance [26,27]. The ratio of (count of NMs)/(count of descriptors) has a cut-off value of 5 (Topliss ratio), which is recommended while regulating to avoid needless complexity, according to the parsimony principle [16,28]. The final number of QSAR descriptors should not exceed six, but when knowledge of the relevance of properties to nanotoxicity is limited, a large number of initial descriptors should be sought [29].

Six studies, out of the 86 gathered, used Genetic Algorithm (GA) for feature selection [30,31,32,33,34,35]. Five of them used Pearson correlation coefficients between pairs of variables to identify those that correlate with the endpoint or correlations among variables to avoid inter-correlations [36,37,38,39]. Few of the studies applied more than one feature selection technique. Papa et al. [40] used GA optimized for Multiple Linear Regression (MLR) models based on ordinary least squares (MLR-OLS) and for support vector machines (SVMs). Using both methods revealed differences in the results related to optimizations for either linear or non-linear approaches. Mu et al. [41] selected optimal descriptors using MLR combined with Pearson and pair-wise correlations, clustering, and Principal Component Analysis (PCA). Clustering and PCA are performed on variables that have significant correlations with observed toxicities. In another study, double cross-validation was additionally used to GA to reduce method-specific selection bias [31]. An overview of feature selection techniques used in the studies is provided in Table 2.

#### 3.2.3. Pre-Processing Techniques

Several techniques exist for pre-processing data in order to make them more suitable for use in computational tools. In the literature reviewed, normalization was used in 18% of cases but other techniques such as one hot encoding, balancing the outcomes, data gap filling, and line notation were used among others.

#### 3.2.4. Normalization and Discretization

In Danauskas and Jurs [22], the base-10 logarithm was applied to limit the range of data while others used normalization of inputs and outputs for increasing accuracy [68]. Another method for homogeneous normalization was taken by References [38,69], where the descriptor pool was pre-processed prior to modeling by autoscaling. This approach is necessary when the data consists of variables with different scaling. A robust z-score is used to normalize the data in order to minimize the influence of outliers [70]. Choi et al. [60] examined several normalization techniques (z-score, min-max, log10) for each attribute in order to reduce the skewness of the data and choose the most appropriate showing that each dataset (and each variable) may require different normalization techniques.

However, there are cases of models where dependent variables are encoded to indicators that only express presence or absence in each dataset instance. One hot encoding is a procedure of converting categorical variables into numeric data to be applied to ML algorithms. These variables take values of 0 or 1 depending on whether a particular nano-feature or experimental endpoint is absent or present [56,71]. Studies used one-hot encoding in models where categories cannot be used, such as in linear regression [60,72]. Within the reviewed articles, one hot encoding was applied in 7% of the studies.

Attribute transformation, such as discretization of numerical attributes and functional transformation, are also commonly performed [73]. Discretization of input was performed in two of the reviewed studies [74,75] based on expert judgment or equal frequency distributions. Discretization is usually performed on classifiers. For binary classification prediction, a cut-off value is used to separate the classes e.g., substances with cellular viability >50% will be regarded as non-toxic. Fourches et al. [76] resulted in binary classification, which transforms the features by splitting at their arithmetic mean. Furxhi I. et al. [18] demonstrated that almost half of the cases derived from the studies in their literature review predicted the outcome in a binary format.

#### 3.2.5. Class Balancing

An issue encountered in both the training and evaluation phases is that hazard classes (i.e., the toxicity classes) are often unbalanced, which means that the number of samples corresponding to one value of the class (e.g., non-toxic) is much higher than the number of samples corresponding to the other values of the class (e.g., toxic) [14]. This imbalance in a dataset, which is an issue particularly prevalent in nanotoxicology, has a negative effect on the algorithm performance. Furthermore, 8% of the studies mention that their dataset had equal outcome classes, while, on the other hand, 4% of studies tackled the imbalance issue by resampling the training dataset. Resampling can be done by applying the Synthetic Minority Oversampling Technique (SMOTE), which is a supervised instance algorithm that oversamples the minority instances using the k-nearest-neighbor (kNN) [60,67,77]. This method balances the dataset by generating more data points. The rest of the studies did not mention class balance issues.

#### 3.2.6. Missing Values

Handling missing values enhances the reliability of the dataset and expands data interoperability, which offers the nano-safety community complete datasets to be used in novel modelling. There are three types of supervised data filling approaches, such as QSAR methods [78], trend analysis, and read-across (interpolation or extrapolation). They are based on different assumptions and, as such, require a different minimum number of data points [5]. Gajewicz [79] mention that existing methods of read-across methodologies are expert knowledge-dependent making the prediction prone to bias. To tackle this issue, they propose a novel quantitative read-across approach based on a simple transparent algorithm for filling data gaps. Several computational tools have been developed for supporting grouping and read-across. Giusti et al. [12] provide an update of existing approaches in NMs grouping while suggesting future recommendations. Other approaches for filling data have been proposed that are dataset-specific. For example, Ban et al. [80] used curve-fitting to calculate missing ages based on the age-weight relationships of different species. While assessing data quality and completeness, nano-specific filling in of missing values using manufacturer’s specifications and/or estimations [60,64] was suggested within the Safe and Sustainable Nanotechnology (S2NANO) (http://portal.s2nano.org/ (Webpage accessed autumn 2019)) database. Furxhi et al. [72] investigated the robustness of several ML tools on generated versions of the dataset by removing values artificially. Recently, an integration of two data gap filling techniques to predict neurotoxicity for non-NMs was implemented, which demonstrated the capacity of integrating methodologies [81].

#### 3.2.7. Molecular Structures’ Codification

An additional issue in the pre-processing of data is the description of molecular structures. Among the most common methods to codify chemical structures are (i) the chemical graph, which represents structures by connection tables, (ii) the linear notations as Simplified Molecular Input-Line Entry System (SMILES), and iii) the de-facto standard chemical formats. SMILES can be obtained by common software like ChemSketch (https://www.acdlabs.com/resources/freeware/chemsketch/ (Webpage accessed autumn 2019)). From the cases gathered, 23 of them use line notations such as SMILES (5 cases), based optimal quasi-SMILES (14 cases), and Improved SMILES (4 cases). Experimental in vitro characteristics and exposure conditions are important variables in the representation of a potential toxicity since the same type of NPs may exhibit diverse effects in different biological conditions. This makes the development of classic QSAR difficult [82]. Toropova et al. [83] suggested a quasi-SMILES approach to represent molecular structures, p-chem properties, and experimental conditions (eclectic data) with NMs [37,82]. The eclectic data are translated into optimal nano-descriptors (the sum of weights of quasi-SMILES) for the outcome prediction and Monte Carlo optimization is used to select the optimal descriptors. Optimal-based SMILES descriptors can be calculated with the International Chemical Identifier (https://iupac.org/who-we-are/divisions/division-details/inchi/ (Webpage accessed autumn 2019)) even though, as noted by References [84,85], SMILES-based descriptors can have some drawbacks for describing endpoints for some NMs and for the interpretability of the models. To overcome the limitations of optimal-SMILES, the Improved SMILES-Based Optimal Descriptors has been proposed [84] as a novel descriptor characterizing structural and chemical properties, which interprets the endpoint more accurately. In a recent study, pseudo-SMILES were tested as descriptors for a random forest method and compared with the linear regression based on an optimal descriptor method [86].

#### 3.2.8. Data Splitting

The final component of data pre-processing/transformation is the splitting of the dataset prior to model implementation. Surprisingly, only 41% of studies mention the technique used and even fewer mention the presence of outliers and how the removal improved model performance. Often such information is omitted as unimportant. Yet such details ensure the reproducibility of a method. Datasets are split into different sub-sets with different roles of (i) a set for training a statistically significant and reliable model, (ii) the test set to measure robustness, and (iii) the validation set to assess predictability of the trained model. Training is done to adjust the model parameters while preventing overfitting. Good predictivity may be achieved for substances significantly similar to those in the training set. A model will perform inadequately for test set substances that differ from the training set. Thus, instances should be selected in a way that ensures that test set substances lie within the properties space defined by the training set [46,87]. In some cases, for further evaluation, unseen datasets are used in order to test the model on data, which are absent in the training and validation step [60,88,89].

Distribution of variables into the training and validation set has an influence on model performance [89]. Several techniques are mentioned in the reviewed studies, including balanced splitting based on one specific variable [90]. Keeping extreme responses (i.e., the highest and lowest range of a variable in the training set [46,84,91]) avoids the risk of extrapolating out of the response range. For this concept, Kar et al. [46] used PCA score plots to confirm that each test set compound was near to or within the chemical space of at least one training set compound. Ghorbanzadeh et al. [55] performed a diversity analysis to check whether structures of the training and test sets represent those of the whole data set. This method enhances model stability and verifies the appropriateness of the external test set to assess the morel predictivity. Some other methods for data division are the k-means clustering method [56,57,71] or the modified Kennard-Stone algorithm where the response vector is replicated *k* (number of descriptors) times in order to enhance the influence of the response on the splitting results [26,48].

Random splitting is the most employed method across the studies, yet different distributions should be tested for the training and validation set to realistically estimate the influence of splitting and, thus, confirm that the final model quality was not random [83,92,93]. Mikolajczyk et al. [94] sorted NPs along increasing values of zeta potential, and then included every third NP in the validation set, using the remaining NPs to form the training set [35]. The same methodology has also been followed elsewhere [48,84]. The methodology used by Puzyn et al. [35] added to the validation set some cases, which do not fall in the range of the training set (validation and reliability testing at the same time). The complete dataset should be provided to potential dataset users, including nanomaterial, endpoints, and descriptor information, together with the clearly defined training and test sets [4].

### 3.3. Model Implementation

The third component of the roadmap is the model implementation of linear or nonlinear models (Figure 1). In this section, the second OECD validation principle—an unambiguous algorithm—requires full model structure and accurate values of all the model parameters to be specified.

**Figure 2:**
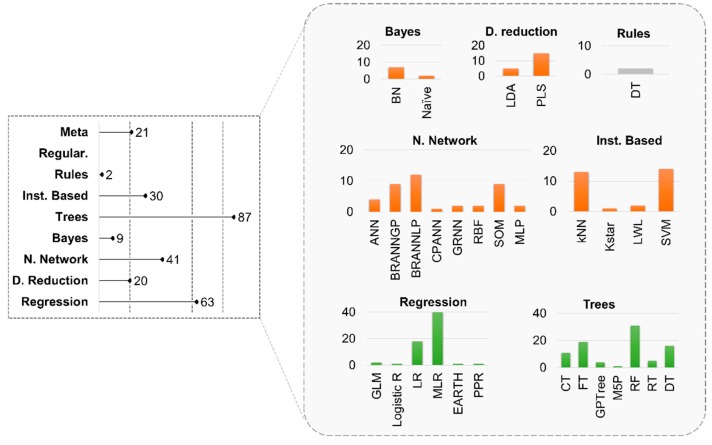
Model (cases) categories, their population (left), and detailed breakdown (right, zoomed box) as extracted from the 273 cases derived from the 86 studies gathered. Instance based (Inst Based), decision tress (D. Tree), Bayesian networks (Bayes), neural networks (N. Network), and dimensionality reduction algorithms (D. reduction).

Of the 86 studies reviewed, 48 performed linear modelling, 51 performed non-linear analysis, and 13 performed both modelling techniques. For each of the studies examined, the combination of model implementation, validation metrics, and the applicability domain were recorded separately, which causes many extractions per study. If, for example, another model was created with the above specifications unchanged, this would be introduced as new case within the analysis. However, if a different dataset is used with the same model, this also leads to a new insertion. This process resulted in the extraction of 273 predictive models (cases) implemented in 86 individual studies (Figure 2).

The most popular data mining ML algorithms can be combined into categories such as (1) rules, (2) instance based, (3) trees, (4) bayes, (5) neural networks, (6) dimensionality reduction algorithms, (7) regression, and (8) meta/ensemble algorithms [95].

In most of the cases, 87 out of 273 trees were implemented (Figure 2) and the most popular algorithm was Random Forest (RF) (31 cases) (Figure 2, zooming box, D. Tree). Functional Trees (FT), Classification Trees (CT), and Decision Trees (DT) followed with 19, 11, and 16 cases, respectively. Random Trees (RT) and Genetic Programming-based decision Trees (GPTree) were used in five and four cases each, whereas only one study implemented an M5 model trees (M5P) algorithm. The application of trees algorithms in the studies to predict diverse endpoints is shown in Table 3.

The DT classifier is a rooted tree where each of its nodes is a partition of the instance space based on gaining information. Horev-Azaria et al. [73] used one of the most common DT algorithms, C4.5, and their implementation starts with cases that are examined for patterns that require categorization of groups. Jones et al. [96] also employ the C4.5 algorithm while Zhang et al. [100] used an RT to associate cytotoxicity with energy conductivity and metal dissolution. They found that the model captured nonlinear dependence between descriptors and cytotoxicity as well as possible interactions. RF is an ML recursive ensemble algorithm based on a combination of independently grown binary decision trees constructed with various samples of a bootstrap [64]. By aggregating the predictions of each tree, the RF algorithm makes forecasts depending significantly on two model parameters. The number of trees and number of variables chosen to be used at each node are rarely mentioned in the studies [80]. Similarly, the RT algorithm divides the output population into groups based on numerical input inequality or categorical input grouping. The input factor and the split criterion are chosen at each branching point to achieve the greatest gain of information [63]. M5P is another algorithm that implements base routines for generating trees and rules [65]. CT starts with a ‘root node’ that contains all objects (i.e., NMs), and then divides by recursive binary splitting into child nodes. Each split is defined by a threshold that takes into account the selected descriptor values at a given stage [105]. The GPTree uses a simplified fitness function from a random population of solutions with repeated attempts to find better solutions through the application of genetic operators. The best trees are chosen by their predictivity [30].

Regression models were the second most commonly used computational tools in nanotoxicology with 63 cases (Figure 2) in the reviewed literature. Multiple Linear Regression (MLR, 40 cases) and Linear Regression (LR, 18 cases) are mostly preferred while the Generalized Linear Model (GLM, 2 cases) is less commonly applied. Logistic R, Multivariate Adaptive Regression Splines (MARS or EARTH), and Projection Pursuit Regression (PPR) appeared only once in the reviewed studies. The application of regression algorithms in studies to predict diverse endpoints is shown in Table 4.

In MLR, the output is expressed as a linear function of the inputs and the degree of descriptors’ influence on output is obtained by the weights of the coefficients. The MLR model is designed to minimize the sum of squares of observed and expected value differences [55]. A descriptor array can be selected using the MLREM sparse feature reduction process. The approach is repeatedly applied increasing sparsity and optimal descriptors are obtained at the starting point of model performance deterioration [56]. One approach for selecting descriptors is to investigate the statistical value of all possible descriptor combinations by using MLR-OLS, which can be performed in QSARINS (http://www.qsar.it/ (webpage accessed winter 2019)) [28]. Partial least squares (PLS) is another method that, due to the lower number of data points, can be used for selected descriptors in a stepwise approach. In the case of PLS, a strict test for the importance of each consecutive element is necessary in order to prevent overfitting [107]. GLM is an extension of conventional regression models, which allows the mean to rely through a relation function on explanatory variables and the response to be any member of a group of distributions called the exponential family. GLM includes statistical models such as LR for normally distributed responses, binary data logistics models, and counting data log-linear models through its general model formulation [60,97]. PPR is a non-parametric approach based on developing a number of non-linear univariate smooth functions. The regression function is then represented by the sum of a finite number of ridge functions. Among the infinite direction of projections, an optimization technique enables a sequence of projections to reveal the data set’s most important structures [40]. The EARTH algorithm constructs models of regression without making any assumptions between dependent and independent variables. The input space is divided into regions with their own regression equation [40].

Instance-based algorithms appeared in 30 of the reviewed studies (Figure 2). The most popular instance-based algorithms were Support Vector Machine (SVM, 14 cases) and k-Nearest Neighbors (kNN, 13 cases). Less frequently used were Kstar and a Locally Weighted Learning (LWL) algorithm. The application of instance-based algorithms in studies to predict diverse endpoints is shown in Table 5.

The kNN method classifies a case in the feature space based on the nearest training instances [62] relying on the similarity principle [40]. Based on weighted majority voting, each case is allocated to the class of the *k*th closest neighbors. The optimal *k* value is selected using distances (generally Euclidian distances) as weighting factors for voting, which characterizes compounds’ dissimilarity in a multidimensional feature space [76]. The *k* value can be selected by a cross-validation method [102]. Fourches et al. [76] used an algorithm combining kNN and a variable selection procedure to maximize model accuracy. SVM is another widely used algorithm for classification and regression. First, SVM defines decision boundaries parting data into different classes [60]. Second, data are mapped in a higher dimensional descriptor space, where a linear representation can better fit [121]. SVM performance depends on kernel function’s shape and on parameters associated with the distribution of learning data. The usual practice to discover the optimal parameters is through the grid search [40]. Three rarely used instance-based algorithms in the field of nanotoxicology are the LWL, Kstar, and Lone-Star. LWL uses an instance-based algorithm for locally weighted learning [96]. In KStar, the class of a test case is based upon the similarity with the training cases, using an entropy-based distance function [65]. The sparse classification Lone-Star algorithm implements optimization methods to overcome issues inherent to nanotoxicity modeling, such as unequal distribution of classes and unknown relationships between inputs. This method, when compared to traditional SVMs, takes advantage of the combined l1-norm and l2-norm SVM’s ability to select a small set of features while ignoring the redundant ones to achieve both the classification goal and the selection of correlated features simultaneously [118].

Neural Networks were applied in 41 cases (Figure 2). In four of the cases, the type of Neural Networks was not provided, but, for the rest, a number of different algorithms were used including neural networks controlled by Laplacian Prior (BRANNLP, 12 cases) or by Gaussian Prior (BRANNGP, 9 cases). Radial Basis Function Neural Networks (RBFNN), General Regression Neural Networks (GRNN), Multi-Layer Perceptron (MLP), and the Counter Propagation neural network (CPANN) algorithms were used in a few instances. The Self-Organizing Map (SOM) algorithm was found in nine cases and the application of the Neural Networks algorithms in the reviewed studies to predict diverse endpoints is shown in Table 6.

Neural Networks were conceived based on functions of the central nervous system and became very popular in discovering relationships between parameters [88]. Different architectures and topologies were noted in the reviewed studies such as RBF, MLP, and GRNN [122]. In MLP, each network is built from several layers connected by weights. These weights are adjusted iteratively during training to reduce network errors [55]. RBFNN are composed of three layers and descriptors are transmitted to the hidden one unprocessed. The hidden layer is made of a few centers whose number and location are automatically defined. Hidden centers’ activation is computed from a transfer function depending on the distance between the center and the cases [40]. GRNN differ from RBF as it forms hidden layers of as many units as the cases. Activations of these units are calculated using a non-parametric estimator for a given object with a probability density function [40]. SOM’s neural networks use unsupervised learners, projecting data onto a two-dimensional display providing an indicator of the degree of similarities between cases. Shorter distances of projection indicate crucial similarities [70]. SOM does not perceive differences between classes and dependent variables [123]. CPANN consists of two active levels of which one is a SOM. Inputs are connected to all units of the map with randomized weights and, for each input pattern, a neuron most similar to the descriptors is determined to enhance the fit in SOM. The neuron is projected in the same place in the second level with adjusted weights between the two maps [40]. In contrast to backpropagation networks, regularized Bayesian networks do not need a validation array to establish when learning should stop. Bayesian regularization controls the complexity of models using Gaussian and Laplacian priors (BRANNGP and BRANNLP, respectively). Laplacian priors prune unrelated descriptors, which leads to robust models by optimizing the sparsity and predictivity [56].

Dimensionality reduction methods were used within 20 of the studies reviewed (Figure 2). Partial Least Squares (PLS) was used in 15 cases and Linear Discriminant Analysis (LDA) was used in five cases. The application of dimensionality reduction algorithms in studies to predict diverse endpoints is shown in Table 7.

LDA is a method that seeks a hyperplane to discrete different endpoints and, as such, LDA is commonly used for dimensionality reduction and classification. Within two of the reviewed studies [124,125], LDA was employed for classification to search for the perturbation model using a forward step-wise procedure. PLS is a fusion of MLR and Principal Component Regression (PCR) and it is one of the most popular approaches in QSARs. Through a linear combination of the original variables, PLS produces a set of components to best represent the output in the descriptor space [40].

Two rules models were found to be employed in the studies reviewed as two versions of Decision Table algorithms (DT) (Figure 2). Rules, as classifiers, include algorithms that dissect the dataset by rules. DT classifiers carry all links between input and output data using the majority of values or the nearest neighbors in the case of unknown data. DT/naive Bayes (DTNB) hybrid classifier splits the attributes into two sub-assemblies: one for DT and the other for naive Bayes [96]. Such Rules models have been used in the studies reviewed to predict only cellular viability.

Twenty-one consensus models with meta/ensemble algorithms were found in the reviewed literature (Figure 2). In this case, ensemble methods unite multiple individual algorithms into a consensus final model to reduce variance and bias or enhance predictivity. The application of meta/ensemble algorithms in studies to predict diverse endpoints is shown in Table 8.

Chau and Yap [121] used a meta algorithm based on the majority voting for the top five out of 2100 individual classifiers. The bagging algorithm generates multiple versions of a predictor, which are then used to generate an aggregated predictor based on multiple versions [65]. In the Decision Tree Boost (DTB), a stochastic boosting is applied repeatedly to increase prediction accuracy. Each function’s output is then merged with weighting to minimize the total prediction error and the loss function in the training set. In the Decision Tree Forest (DTF), independent trees are developed in parallel without interacting. Learning sets are then drawn randomly with replacement from the training dataset, which produces different models to predict the entire dataset. The models are then aggregated. The DTF uses data rows left out to validate the model without the requirement of a separate data set. Kovalishyn et al. [102] built an ensemble of backpropagation neural networks while applying the kNN method to determine the local correction of the Associative Neural Networks (ASNN). Their ASNN ensemble included 100 networks.

While *Bayes* models offer visual representation of the variables’ connection and perform well with missing values, only nine cases were found to be applied in the reviewed literature including seven of which were Bayesian Networks (BN) and two were Naïve Bayes (Figure 2). BN are graphical models that encode probabilistic relationships among random variables. The distribution of these variables with respect to the categories is used to assign a probability of pertinence to each category. The accumulated pertinence probability across all nodes, which are presumed independent, are used for categorization. The application of Bayes algorithms in studies to predict diverse endpoints is shown in Table 9.

BN can be fed with varying datasets that may lack data through their ability to iteratively refine prediction as novel knowledge becomes accessible [128]. The structure of the model is optimized using data for every node and the conditional probability tables to determine the ideal configuration of the nodes’ interactions [127]. Naive Bayes uses posterior probability to predict the target attribute’s value. The classifier tries to find the value that maximizes the conditional probability of the target attribute by using a given input [96]. Assuming that, for a given outcome, input attributes are independent, naïve Bayes is easily implemented since the calculation of the probability is straightforward based on the Bayes theorem by counting the frequency of values and combinations in historical data [73,121].

**Figure 3:**
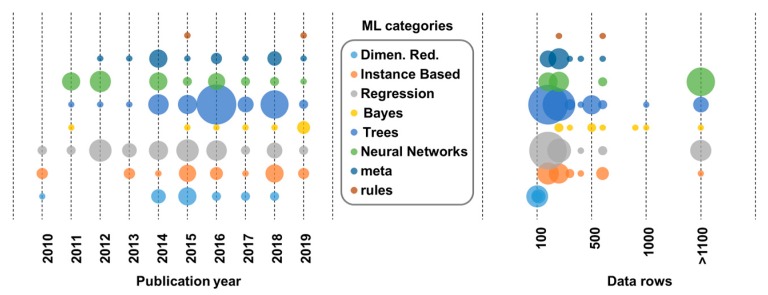
Machine learning categories used over the last decade (**left**) and their relation with data size samples (**right**).

**Figure 4:**
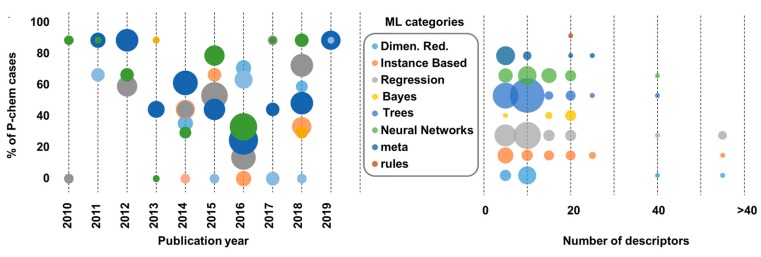
Machine learning categories vs. number of descriptors (**right**) and vs percentage of p-chem data cases over the years (**left**).

In Figure 3 and Figure 4, we demonstrate the different machine learning categories used over the last decade and their relation with the data size samples (Figure 3). In addition, we show the categories used in relation to the number of theoretical descriptors used in the final model and the percentage of the nano-specific p-chem properties over the years (Figure 4).

### 3.4. Model Validation and Applicability Domain

The fourth OECD principle includes goodness-of-fit, robustness, and predictability measures aiming at distinguishing the elements between internal and external validation. As stated in the OCED document [19], no absolute predictivity calculation is sufficient for all purposes and varies depending on the statistical methods used in the analysis.

#### 3.4.1. Goodness-of-Fit

Of the studies reviewed, 78% report internal validation with calculation of performance metrics to demonstrate the goodness-of-fit, which is a measure of how well the model accounts for variability in the training set’s response. The quality of regression can be assessed by the squared correlation coefficient (*R*^2^) [54] or the standard error of estimation (SEE) [57]. Only models with a higher *R*^2^ than the thresholds defined in previous studies should be considered acceptable [8]. Furthermore, the adjusted R-squared (Radj^2^) value can also be calculated in order to prevent over-fitting [38]. Radj^2^ is interpreted in the same way as the *R*^2^ value except that it takes the number of degrees of freedom into account. The equations of the above metrics can be found in Supplementary Materials. A number of studies did not report internal validation, as they focus on more demanding metrics like robustness.

#### 3.4.2. Robustness

The term ‘robustness,’ in this case, refers to the stability of model predictions when a perturbation is applied to the training set and 69% of the studies reviewed provide some information about model robustness. Commonly, robustness evaluation for ML is done through a *k*-fold cross-validation, by randomly dividing the data set into k subsets, and then computing the average performance across all *k* trials [63]. Root Mean Square Error (RMSE) may be used to specify the model’s calibration ability. If two regression models have similar RMSE, F-values (the ratio between explained and unexplained variance) and P-values (the probability of finding the observed or more extreme results) can help determine the model of choice [22,129]. Robustness metrics such as squared cross validated correlation coefficient (Q^2^), leave-one-out cross-validation coefficient (Q^2^_LOO_), and leave-many-out cross-validation coefficients (Q^2^_LMO−10%_ and Q^2^_LMO−25%_) are popular robustness indicators [46,47]. To avoid the possibility of overestimation by using only leave-one-out cross validation, a bootstrap procedure (Q^2^_Boot_) is suggested [23] and is mainly suitable for a limited number of training cases [50]. These approaches systematically take out data points from the training set, reconstructing the model, and then predict the left-out data points. The leave-many-out approach remove a different number of values from the data set (10%, 20%, 25%, or 50%), depending on the size of the dataset even though there is no rule-of-thumb as to the percentages one should apply for cross validation or data split. Besides Q^2^_LOO_, the root-mean square error of cross-validation (R^2^_CV_) can be calculated [38,94]. The minimum criteria for a successful QSAR model is *R*^2^ ≥ 0.6 and Q^2^_LMO_ of ≥ 0.5 [84], whereas training and the test set *R*^2^ value difference should not exceed 0.3 [56].

To further assess the robustness, standard deviation based on predicted residual sum of squares (PRESS) can be calculated [55], which, in small values, suggests model insensibility to single data points. For binary classification problems, validation metrics derived by the confusion matrix, for both goodness-of fit and robustness, include accuracy, sensitivity, specificity, and the correct classification rate (CCR) [76]. Across these approaches, the classification models are regarded as acceptable if CCR_CV_ ≥ 0.6 and CCR_test_ ≥ 0.6 [76]. Other metrics include the F1-score, Matthews correlation coefficient (MCC), discriminant power, and the Receiver Operating Characteristic (ROC) curve. The ROC graph can be applied to show, comparatively, two-group classification models’ predictive capabilities. The equations of the above metrics are provided in Supplementary Materials.

#### 3.4.3. Chance Testing

Where there is a large number of variables, such as is often the case in nanotoxicology, some variables are likely to be chosen by chance. To verify model robustness, a y-randomization permutation test is used to avoid ‘‘correlation-by-chance’’ possibilities confirming the model’s statistical significance [76]. Within the y-randomization permutation test, the values of output are mixed and the correlation coefficient is determined. The scrambled-output R^2^ is compared to the model’s R^2^. The model is not reliable if the two values are identical [40,44]. Similarly, the ‘‘true’’ model can be characterized by calculating the values of RMSE and RMSE_CV_ [34]. Monte Carlo can also be used, whereby the dependent variable is randomized and the models rerun [22], as well as ensuring model’s Q^2^_CV_ statistically significance value [54], the CCR acceptance thresholds [76], or its prediction accuracy [30]. QUIK (Q under influence of K) rule [28], which is a basic criterion that optimizes the ranking of the best features combinations, enables high predictor collinearity models to be rejected [40]. While all previous studies mentioned compare the values of the “true” and random models, a new metric is used elsewhere [46,53]. The randomized model’s squared average correlation coefficient ($R_{r}^{2}$) should be lower than the original model’s *R*^2^. Another metric (based on the $R_{r}^{2}$) $cR_{p}^{2}$ can range from 0 to 1 with a $cR_{p}^{2}$ value greater than 0.5 defining what can be considered an acceptable model. The equations of the above metrics are provided in Supplementary Materials. Models should be selected for further external validation if they can predict the training set (goodness-of-fit) and the test set (robustness).

#### 3.4.4. Predictability

The use of external validation is being increasingly recommended by researchers and authorities for the assessment of model reliability. Internal validation provides an optimistically skewed estimate of the real predictive potential [14]. In addition, 60% of the reviewed studies performed some form of external validation. However, this does not indicate that the reported statistics are sufficient to fully evaluate model performance. In addition, using more than one validation metric to calculate the accuracy of the model prediction is always advantageous [29]. The quality of the resulting models can be evaluated by the mean squared error (MSE) [63] and the Q^2^_ext_ value [42]. A standard error of prediction (SEP) or its deviation (SDEP) and slopes k have also been used [130]. SEP is the calibrated error to the degrees of freedom between predicted and measured endpoints [57]. Predictability can also be assessed through the root mean square error of prediction (RMSEP) [41]. Mean absolute error (MAE) is regarded as a straightforward error determinant [25] and QSARs should meet the criteria: MAE ≤ 0.1 × (train set range) and MAE + 3σ ≤ 0.2 × (train set range). Concordance correlation coefficient (CCC) is a restrictive parameter for predictability [95,126]. The $r_{m}^{2}$ metric provides the stringent external validation criterion at a given threshold value, which can be adopted for regulatory processes [131]. Likewise, it is possible to use $\bar{r_{m}^{2}\left(LOO\right)}$ for the training set [46], which may reflect the model’s external validation characteristics [53]. Among the metrics mentioned, $r_{m}^{2}$ displays significantly different values from other measures including CCC, which is the most confident [131]. For binary classification, the sensitivity, specificity, accuracy, and ROC curves can be calculated [73,104]. Some of the reviewed models within the peer-reviewed literature did not demonstrate any validation metrics at all [123,127].

#### 3.4.5. Ranking of Classifiers

Roy et al. [132] proposed a composite score of predictions using a reliability indicator. This is a tool based on absolute prediction errors to rank the quality of predictions. The tool ranks the models into good, moderate, and bad, using three criteria. However, the tool is presently valid only for MLR models. Furxhi et al. [72] proposed a composite score based on a Copeland index to rank classifiers according to their performance on diverse datasets, validation stages, and performance metrics. Tamvakis et al. [133] proposed a dissimilarity performance index based on their voting performance to recommend the optimal ensemble combination. A variety of different datasets were used in this scenario to evaluate the relationship between voting results and dissimilarity measurements. Tsiliki et al. [134] proposed an integrated, fully validated procedure framework, which implements multiple models and uses cross-validation averages for model selection.

#### 3.4.6. Applicability Domain (AD)

**Figure 5:**
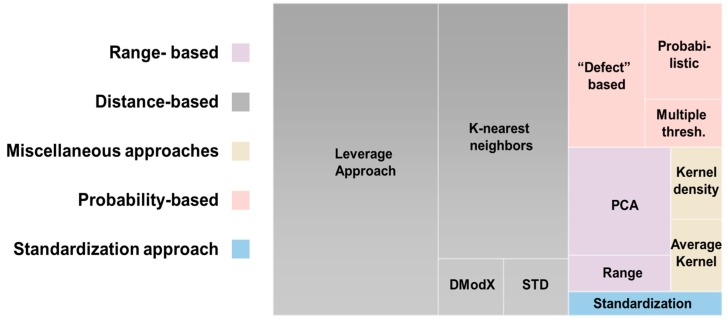
Methods determining the applicability domain of a model.

The descriptor space in which the model was trained is essential and defining the applicability domain (AD) is required as the third OECD principle of validation. Predictions extrapolated outside the model’s AD may be less accurate [76]. While model AD is a dynamic area of modelling analysis, there is no universal AD definition technique. Usually, the AD definition is based on an arbitrarily outlined distance between the analyzed NM and the training set compounds [135]. Several methods for determining the AD exist [136] as seen in Figure 5 and approximately half of the studies reviewed define the AD of their models.

As used in three of the studies reviewed [28,40,111], the AD of classifiers can be checked by PCA using the descriptor correlation matrix to symbolize the training and prediction distribution set within the used model’s space. Consideration of the descriptors’ ranges is a straightforward way to characterize the AD. This method assumes that the descriptor values obey a normal distribution and, therefore, could be inaccurate if this presumption is breached. Singh and Gupta [126] used different approaches to evaluate the AD with the first based on the ranges of descriptors and the second based on the leverage approach. The second most common method is based on the leverage approach and Williams plot (Figure 5). The leverage approach offers an inspection of multivariate normality providing a measure of a compound’s distance from the model’s space centroid. Williams’s plot (standardized cross-validated residuals vs. leverage values) can be used to visualize a QSAR’s AD and check the existence of outliers [23]. It is stressed that the leverage in the William graph quantifies only linear similarity. Therefore, this approach is only applicable to linear regression models [14]. In addition to AD based on Williams plot, Euclidean-based AD can be used to detect the outliers. Determination of the AD for non-linear models can be accomplished by the average kernel similarity [50]. AD can also be determined based on a kernel density estimator, which is a non-parametric probability density distribution-based method [137]. Non-parametric techniques have the capacity to detect empty spaces within and to generate regions around the interpolation space boundaries to reflect the distribution of data.

AD’s distance approach (e.g., Euclidean, Manhattan, and Mahalanobis) is based on calculating the distance of a test compound and a defined point in the model’s descriptor space. The prediction is inaccurate if the distance exceeds the threshold [61]. The benefit of this approach is that, by drawing isodistance contours in the interpolation space, confidence levels can be associated with the AD. The disadvantage is, once again, the assumption of a normal distribution for the underlying data. Xia et al. [138] verified the AD of their models by the leverage approach versus the Euclidean distances measured by the jackknifed residuals. If a compound’s jackknifed residual is greater than 2.5 times, the compound will be treated as an outlier.

Sizochenko et al. [104] estimated the AD based on minimum-cost-tree of variable importance values in the space of descriptors while Kar et al. [46] used diverse approaches to assess AD, such as the leverage approach and distance to the model in X-space (DModX) (Figure 5). The DModX approach is usually applied for PLS models and the basic theory is that Y and X residuals have a diagnostic value for model reliability. Since there are a number of X-residuals, a summary is required and this is accomplished by the standard deviation of the X of the matrix corresponding row. Kovalishyn et al. [102] used the ensemble predictions standard deviation (STD), which correlates with predictions’ accuracy. The method shows that the prediction is more likely to be unreliable if dissimilar models give significantly dissimilar predictions for a case and STD is preferably used as a model uncertainty estimator.

Toropova and Toropov [114] suggested the idea of “defect” to the AD of quasi-QSARs (Figure 5). The quasi-SMILES defect is characterized as the sum of each quasi-SMILES component defect and is calculated according to probabilities [37]. Another method is the multiple threshold method used by Chau and Yap [121], which is a method originally proposed by G. Fumera [139]. The AD can also be calculated by the standardization approach, which is a straightforward method proposed by Roy et al. [140] for terming the outliers and for identifying compounds outside the domain (validation and prediction set) [105]. Compared with the leverage strategy, the proposed method works well. The method does not, however, consider inter-correlation between descriptors and does not consider descriptor relative contribution.

Twenty-six out of 86 studies fully validated their models and demonstrated the AD as shown in Table 10. The minimum amount of data rows was 6 data points and the maximum was around 7000 data points.

## 4. Discussion

We provided an overview of data pre-processing techniques, model implementation, validation, and applicability domain of ML methods used in predicting human health and ecotoxicological hazard endpoints. We focused on recording methodologies rather than a critical assessment of the available tools, leaving the fifth OECD principle, which is a mechanistic interpretation, out of the scope of this study.

### 4.1. The Framework

Variable selection was commonly used in the articles reviewed with almost 50% of the studies using a feature selection method. Since most of the models developed have been based on implementing classic QSARs (i.e., using generated theoretical descriptors), initial feature reduction and selection were required. Various metrics of variable correlation may give different results and descriptors that seem highly associated with one method may not be redundant. Selecting the most appropriate descriptors is always based on the process (e.g., choice of GA or ERM). GA showed great performance among the different methods for feature selection, while ERM was superior in some cases as a total search algorithm and, thus, less reliant on the initial set of descriptors. Such cases make the selection of statistical features a dynamic research area [4]. We recommend either a combination of different feature selection techniques to evaluate possible differences in the results, or, more efficiently, an integration of techniques proven to outperform individual methods and mitigate any method bias [143].

Different models that use measured p-chem properties and experimental data, including biological data, exploit all the features since those properties are nano-specific [60,77]. QSAR-perturbation models, in addition to classical QSARs, make use of all available descriptors by generating several pairs of variables using the moving average approach [122,125]. Contrary to the feature reduction problem of theoretical generated descriptors, using nano-specific properties comes with data lacunas and the need for more descriptors. Properties like size, surface area, crystallinity, composition, solubility, shape, and surface reactivity affect NP biological interactions and should be represented explicitly or implicitly by proxies in models [144].

Several studies have developed quasi-QSARs using line notation methods, such as SMILES, to represent the structure of a molecule in a character string. This codification enables using the SMILES-specific models to classify non-SMILES descriptors. It should be noted that the application of a mixture of SMILES generated by different software packages is improper [93]. Optimal SMILES-based models outperform models based on optimal descriptors, since combining global attributes and SMILES components provides more information on the molecular structure than traditional descriptors [130].

Class imbalance reflects an unequal distribution of class values within a dataset and poses a challenging problem because classifiers exhibit biases of the results. This has been rarely accounted for properly during training [60,67,72,74]. The most common technique used was SMOTE. SMOTE looks at the feature space for the minority class data points and generates new points considering its k nearest neighbors. The class imbalance not only affects model performance, but it also affects features correlation. Once a balanced dataset is attained, feature correlation becomes more accurate.

Regarding data normalization, it is advisable to select a different normalization technique (z-score, min-max, log10) for each variable, according to the skewness of feature data [60].

Single random splitting was common across the studies. However, our research shows clearly that multiple random training and validation distributions should be examined to investigate the influence that the split may have on attribute distribution and to ensure randomness. Even though there is a no general rule-of-thumb for setting a splitting point, the 80/20 was the most commonly used ratio, often referred to as the Pareto principle [145]. If the dataset is not balanced, the data should be stratified before splitting. However, such information is often not reported. Since most of the datasets used in nanotoxicology are quite small and splitting may hinder a satisfactory variance in the estimates, k-cross validation should be performed. Moreover, after splitting, correlation between the data in the training or test set should be minimal and the test data should be contained within the chemical space identified by the training data. The latter can be covered by using the PCA score plot or diversity analysis [46,55] and investigation of multiple splitting can be performed following the methodology from Puzyn et al. [35], which ensures that validation data are evenly distributed within the range of toxicity of the training dataset. The complete dataset of substances, endpoints, and descriptor values should be annexed in each analysis, along with clearly defined learning and test sets [4].

In order to evaluate model performances, it is essential to provide proper metrics and statistics and 78% of the reviewed studies presented evidence of internal validation. Almost half of the studies investigated robustness performance and 60% of the studies performed external validation. k-fold validation provides a superior estimate of the generalization error since it is less affected by overfitting [73]. *R*^2^ can be artificially increased by adding parameters while Q^2^_cv,_ decreases when a system is over-parameterized, which makes Q^2^_cv_ a more accurate measure of models’ predictability [54].

The Matthews Correlation Coefficient (MCC) is a good choice to demonstrate any biases in the dataset and even in the presence of imbalance classes [146]. MCC is equivalent to the Pearson Correlation Coefficient for binary variables [147] and has been selected as an evaluation metric for micro-array-based predictive models by the MicroArray Quality Control (MAQC) Consortium [148]. From the gathered studies, only six used MCC as a performance metric [77,118,121,122,124,126].

Table 10 presents the studies that, in compliance with OECD principles, applied measures of robustness and predictivity validation, and estimated the applicability domain. It should be noted that choosing the right metric depends on data distribution and splitting, and a combination or aggregation of metrics should be preferred. Statistical hypothesis testing could be performed to investigate whether the difference between the ranked models is statistically significant. Besides the statistical methods already used and reported in the Methods section, Rodríguez-Fdez et al. [149] compiled techniques specialized for ML algorithms and made available online, which can be readily applied for comparing classifiers.

Overall, there was an inadequate assessment of the uncertainty and sensitivity of the methods in the studies collected. A thorough study of uncertainties and areas of variability, bias, and influence of QSAR models is presented in the work of Cronin et al. [150]. Based on their analysis, the authors provide uncertainty assessment criteria for QSAR evaluation classified as relevant to Model Creation, Description and Application. The first two themes follow and extend the OECD validation principles, while the third one complements the assessment on issues of practical use of a model, its reproducibility, and fit-of-purpose. Only a portion of the 49 criteria suggested by the authors are addressed by several nanotoxicological studies.

### 4.2. The Algorithms

Within the reviewed studies, trees, neural network, and regression algorithms were abundant compared to rules, bayes, or meta algorithms. Trees algorithms were used in most of the cases with RF being the most favorable approach applied. Trees are simple to understand and interpret and can be used even with small datasets. They are unaffected by data shortcomings that result in small changes of the outcome and are associated with high dimensionality, correlated variables, and missing values [66]. RF has been demonstrated to be ideal for rigorous meta-analysis of complex and heterogeneous data [64]. Helma et al. [36] note in their study that, with the exclusion of p-chem/proteomics descriptors, the RF model performed better than PLS and weighted average models. They showed excellent predictivity with small or large datasets, which performed well even with missing values. Furxhi et al. [72] demonstrated that RF ranks first among individual classifiers and compete with meta-algorithms. RF is highly tolerant of overfitting, as it combines a number of simple models and has the ability to deal with special issues, such as descriptors counting higher than observations [40]. RT has shown great results in the sense of parsimony, but are more susceptible to biases relative to RF. In addition, RF has the benefit of fully investigating parameter’s values as opposed to RT, which usually includes a small subset of the data set. RF is also less prone to data vulnerabilities due to overrepresentations in datasets, which cause instances to appear influential. RF’s randomized selection ensures analysis of all variables [63].

DT easily handles feature interactions and they are non-parametric but some drawbacks is the non-support of ongoing learning. Therefore, trees must be rebuilt with each inclusion of new data. They easily overfit and can also take up a lot of memory.

Regression models were the second most commonly used usually as MLR and LR. As a result of their simplicity and uncomplicated interpretation, MLRs are used widely. Compared to other models that cannot be visually presented, e.g., RF and MLR can be prioritized due to its transparent structure [87]. PLS can be used instead of MLR in cases of smaller data sets, assuring that strict component significance tests are applied to avoid overfitting [107]. PLS is suitable when there is descriptors co-linearity, while the parameters of the model, such as weights, regression coefficients, selectivity ratios, and the scores of variable importance on projections can be used to measure variable significance [38]. Logistic regression is generally based on the hypothesis that there is a relationship between dependent and independent variables. When the assumption is not true, algorithms that do not make such an assumption, e.g., instance-based algorithms, outperform logistic regression models [121].

The most popular instance-based algorithms were SVMs and kNN. The kNN method is a popular read-across strategy as it requires few similarities and is less computationally intensive and easier to implement than SVM. However, in the case of the complex problem of multi-label variables, kNN may take longer to find the k nearest neighbors. In such cases of very high-dimensional spaces, SVM is more appropriate. SVM is highly accurate, insensible to overfitting, and can work well with a suitable kernel even if data cannot be linearly separated in the feature space. However, SVMs are hard to adjust and interpret. SVMs are memory-intensive. Similar to DT and LR, kNN is highly influenced by the size of the available data set and more data may help in making the model more consistent and accurate.

Bayes can be restructured as new scientific data becomes available and contemporary research grows, which enhances underlying assumptions in the construction of the initial model [77]. BNs provide the capacity to merge different common (i.e., experimental data) and non-traditional (i.e., expert judgment, simulated data) knowledge bases into the BN parameterization process. This is attractive in data-scarce environments such as the nanotoxicology arena [75]. BNs based on Bayes’ theorem are relatively simple to build and particularly valuable for large data sets. Naive Bayes is recognized to outperform sophisticated methods of classification along with simplicity, and is also a good choice when memory resources are a restrictive factor. It should be noted that Bayes classifiers use categorical data. Therefore, numerical attributes have to be converted to each category by replacing numerical data by their corresponding bin-ranges. The finer a range splitting, the more precise the representation of the data and the more demanding computationally the model is. The uncertainty introduced by grouping the data into bins should be addressed when Bayes models are implemented.

Meta algorithms can improve model predictivity and reduce overfitting. The need, though, for developing models that include a directed causality between the nanoform and its toxic activity is clearly stated under the fifth OECD QSAR validation principle and has discouraged the meta-algorithm application. On the other hand, although lacking a mechanistic interpretation, RF has often been used for combining robustness, resources efficiency, and simple parameterizations.

A neural network structure is not easily readable. Their trained parameterizations are hard to comprehend and they can be very resource and memory intensive. Bayesian regularized networks create models that are reasonably insensitive to the number of hidden layer nodes, which makes architecture optimization effortless [58]. A lot of research has been dedicated to ANN especially in pattern recognition, and the advances in the algorithms have been ported to nanotoxicity applications. Due to their potential high complexity, ANN can accommodate plenty of data and still achieve high accuracies with evident computational cost. However, while ANN can accommodate large datasets, small datasets, on the other hand, render ANN prone to overfitting.

### 4.3. Challenges and Perspectives

When only small data sets are available, models that have few parameters (low complexity) and/or a prior strength should be used and, in this case, a ‘prior’ can be interpreted as any assumption on how the data behaves. In linear regression, for instance, the number of parameters can be easily adapted and the models assume only linear interactions. In simple terms, Bayesian models such as Naive Bayes deal with a few parameters and a direct way to adjust their prior.

Neural networks were the only ML algorithm reviewed using more datasets exceeding 1000 cases than smaller ones (Figure 3, right). Trees and regression models were used, as expected, to handle smaller datasets. It is worth noting that Bayesian networks, although not frequently preferred, have been used in all ranges of dataset sizes. Given the present scarceness of nanotoxicity data, the use of effective modelling of small datasets is required [29]. However, even the best algorithm trained with small datasets can be defeated by less sophisticated algorithms trained with more data [151]. Datasets and/or databases integration can be a solution to data scarcity, which generates new hypotheses and knowledge [152]. Karcher et al. [152] highlighted the importance of data integration in nanotechnology and provided recommendations for advancing integration.

Regarding the number of descriptors in the models reviewed (Figure 4 left), ~75% of the studies used less than 10 descriptors, which reflects computational limitations or a lack of data. In Furxhi I. et al. [18], a thorough analysis of the data issue in computational nanotoxicology is provided, stretching from missing data to experimental protocols and concepts. There is a shift in ongoing research toward monitoring, identifying, and quantifying p-chem properties of nanoforms (Figure 4, right). This is evident both in terms of increases in the base expectation of particle characterization in academic journals and also in the objectives of new projects such as the Horizon 2020 project *Nanocommons* (https://www.nanocommons.eu/ (Webpage accessed autumn 2019)), where Work package 5 is focused on learning from raw experimental data, such as microscopic images or spectral data.

There are no specific trends revealed by breaking down the number of cases by the ML technique used over the last decade (Figure 3, left), other than those of trees and bayesian networks that started gaining popularity during the last five years, and neural networks and regression maintaining a longstanding presence in the field. Targeting multiplicity and arbitrariness in model implementation, the EU funded Horizon 2020 project *NanoSolveIT* (https://nanosolveit.eu/ (Webpage accessed autumn 2019)) aims at delivering a validated, sustainable, multi-scale nano-informatics strategy, via OECD-style case studies for the assessment of potential adverse effects of NM on human health and the environment. The project includes the development of cost effective nano-informatics tools and models based on Artificial Intelligence for the prediction of crucial NMs functionalities and adverse effects from descriptors and physical characteristics of NMs.

Nanoforms toxicity databases are available at a developmental stage and data obtained from research studies originate from different experimental procedures. Furthermore, the development of reliable data sets from a computational perspective requires that data be sufficient to allow splitting after assessing its accuracy and suitability specifically for computational use [8]. Knowledge-based expert systems often refer to data-driven modeling. Those systems of expertise derive information from both literature and databases and are considered important tools for predicting toxicity. Considering the lacunas and variations in the accessible nanotoxicity data, knowledge-based expert systems can be a valuable approach for QSARs with a kind of “text data mining” capacity constantly capturing new knowledge that emerges in the literature and knowledge-transfer extracting knowledge from diverse fields [14,29].

## 5. Conclusions

This review of the current state-of-the-art ML computational tools in nanotoxicology, addressing both human health and eco-toxicological endpoints, identified several models that provide prediction to numerous nanotoxicological outcomes. The main conclusions are:

- a variety of ML algorithms have been used during the last decade with non-linear modelling gaining popularity;
- linear regression is still a popular method, enriched with nonlinear techniques;
- there is a clear shift from theoretical descriptors and traditional QSAR modelling to models incorporating nano-specific features, even though there is limited consensus on which features must be considered;
- there is great diversity in data pre-processing techniques depending on datasets and the ML algorithm chosen;
- there is little technical convergence in pre-modelling stage methods compared to model implementation and validation;
- there is, in general, a lack of justification of model selection. There is also little justification on the validation metrics choice.

Implementing ML in nanotoxicology comprises a very active and diverse collection of ongoing efforts. While still in their infancy toward a scientific accord and subsequent guidelines and regulation adoption, ML applications are transforming our ability to predict toxicities from nano-features and experimental conditions. Research in progress on fragmented data integration and curation, in compliance with in silico methods, is expected to enable method testing and an inter-comparison and lead to method standardization.

## Figures and Tables

**Table 1:** Review protocol.

Subject	Description	Subject	Description
**Databases**	Google Scholar, Elsevier (Scopus and ScienceDirect), Web of Science and PubMed	**Exclusion criteria**	Studies predicting nano-properties, environmental outcomes, pharmacokinetic modelling
**Keywords**	nanoparticle, nanomaterial, in silico, computational, machine learning, model, nanotoxicity	**Publication type**	Peer-reviewed journals and reports
**Search files**	title, abstract, keywords	**Time interval**	2010–2019

**Table 2:** An overview of selection techniques used in the reviewed studies.

Feature Selection	Description	References
**Principal component analysis (PCA)**	Widely used for analysis of multivariate datasets applies transformation of observations to PC space with an objective to minimize the correlation and maximize the variance.	[42,43]
**Partial least squares (PLS) with plots**	Applied to predict a set of dependent variables from independent ones, finding the best correlation between them by extracting a number of latent variables preserves information. PLS reveals the most important variables and determines the influence of inputs on output. Star plots produce qualitative selections regarding descriptor importance.	[38,44,45,46]
**Jackknifing**	A resampling technique preceding bootstrap that estimates variance and bias.	[47]
**Genetic algorithm (GA)**	GA is applied to select from descriptors the best combinations for highest predictivity. Based on biological evolution, GA performs function optimization stochastically.	[23,27,30,31,44,45]
**Enhanced replacement method (ERM)**	ERM is a full search algorithm that avoids local minima and shows little dependency on the initial set of descriptors. As such, it can be preferable to GA, depending on the case.	[26,48]
**Genetic function approximation (GFA)**	The GFA method finds out the most frequent descriptors in a large set. The GFA smoothing factor controls the number of independent variables and is varied to determine the optimal number of descriptors.	[32,46]
**Sequential forward selection (SFS) and sequential forward floating selection (SFFS)**	At each step of the selection process, the descriptor that led to the highest model performance is retained until a specified number of descriptors are selected. As an extension to SFS, after each forward selection step, SFFS conducts backward elimination to evaluate descriptors that can be removed.	[49,50,51,52]
**Multiple linear regression (MLR) feature selections**	(1) In MLR, a set of models is examined for stability and validity. (2) One of the most commonly used methods is the GA-MLR. GA deals with optimizing the nonlinear parameters, while the linear ones are calculated by MLR. (3) MLR with expectation maximization (MLREM) is an iterative method that increases the dataset sparsity varying the values of control hyperparameters. The descriptors are selected at the iteration beyond which the model quality is significantly reduced. (4) MLR models based on ordinary least squares (MLR-OLS).	[53,54,55,56,57,58]
**Attribute significance-Importance**	(1) Evaluation and ranking for selecting descriptors based on the variance reduction or entropy as a measure of information gain. (2) Relative importance quantitative estimation based on information or entropy gained from the models. The advantage of the importance based on model information is that it is closely tied to model performance. (3) Comparison of leave-one-out (LOO) errors. Dependences and complements among multiple attributes may not be accounted for by LOO. (4) Worth of an attribute e.g., RELIEF algorithm estimates attributes according to how well their values distinguish among similar instances. (5) Weights calculation by chi-square. A nonparametric statistical technique that compares the observed distribution of frequencies with an expected theoretical one.	[59,60,61,62,63,64,65,66,67]

**Table 3:** Endpoints predicted by trees category extracted from the studies gathered.

Reference	NMs Category	Output	Reference	NMs Category	Output
[80]	Carbon-based, Metal, Metal Oxide, Quantum Dots	Accumulation, reproductive toxicity	[64]	Metal, Metal oxide	Cellular Viability
[63]	Carbon-based	Total protein, Macrophages, Membrane integrity, Neutrophils	[73]	Metal	
[65]	Metal, dendrimer, metal oxide, polymeric	Aggregated	[96]	Dendrimers	
[97]	Metal, Metal oxide, Quantum Dots		[98]	Carbon-based	
[30]	Metal, Metal oxide	Aggregated, Exocytosis, Viability	[37]		
[36]	Metal	Cell association	[66]	Carbon-based, Metal, Metal Oxide, Polymeric, dendrimers, Quantum Dots	
[40]	Metal		[67]	Metal	
[30]	Metal Oxide	Cellular uptake	[99]	Quantum Dots	
[100]	Carbon-based	Dose-response	[25]	Metal Oxide	
[40]	Metal Oxide	Membrane integrity	[60]		
[101]	Metal, Metal oxide	Minimum Inhibitory Concentration (MIC), Viability	[77]		
[102]			[87]		
[103]	Carbon-based	Mitotoxicity	[104]		
[105]	Metal Oxide	No-Observed-Adverse-Effect concentration (NOAEC), Oxidative stress, Protein carbonylation			

**Table 4:** Endpoints predicted by regression tools extracted from the studies gathered.

Reference	NMs Category	Output	Reference	NMs Category	Output
[32]	Carbon-based	Aggregated, Viability	[60]	Metal Oxide	Viability
[56]	Metal Oxide	Apoptosis, Cellular uptake	[82]		
[58]		Apoptosis	[72]		
[50]	Metal	Cell association	[34]		
[40]			[46]		
[103]	Carbon-based	Mitotoxicity	[57]		
[55]	Metal Oxide	Cellular uptake	[31]		
[50]			[41]		
[89]			[84]		
[33]			[106]		
[58]			[35]		
[107]	Metal Oxide, Quantum Dots	Inhibition Ratio, Viability	[108]		
[109]	Carbon-based	Mutagenicity	[110]		
[57]	Metal Oxide	Membrane integrity, oxidative stress	[83]		
[111]	Metal Oxide	Membrane integrity	[112]		
[28]			[69]		
[113]			[97]	Dendrimers	
[114]			[115]	Metal	
[116]			[117]		

**Table 5:** Endpoints predicted by instance-based tools extracted from the studies gathered.

Reference	NMs Category	Output	Reference	NMs Category	Output
[118]	Carbon-based	exposed/not exposed groups	[60]	Metal Oxide	Viability
[76]	Metal, Metal oxide, Quantum Dots	Aggregated, Cellular uptake	[98]	Carbon-based	
[119]	Metal	Aggregated	[72]	Metal Oxide	
[111]	Metal, dendrimer, metal oxide, polymeric		[96]	Dendrimers	
[36]	Metal	Cell association	[67]	Metal	
[111]			[61]	Carbon-based	
[40]			[120]	Metal Oxide	Dose-response
[50]	Metal Oxide	Cellular uptake	[65]		
[62]			[28]		Membrane integrity
[65]	Metal, dendrimer, metal oxide, polymeric	Mortality rate	[102]	Metal, Metal oxide	MIC, mortality rate, viability

**Table 6:** Endpoints predicted by a neural network extracted from the studies gathered.

Reference	NMs Category	Output	Reference	NMs Category	Output
[122]	Metal, Metal oxide, Quantum Dots	Aggregated	[88]	Polymeric	Viability
[70]			[60]	Metal Oxide	
[123]	Metal, Metal oxide		[72]		
[56]	Metal Oxide	Apoptosis	[57]		
[58]	Quantum Dots		[70]	Metal, Metal Oxide, Quantum Dots	
[40]	Metal	Cell association	[57]	Metal Oxide	Membrane integrity
[56]	Metal Oxide	Cellular uptake	[28]	Metal Oxide	
[55]			[103]	Carbon-based	Mitotoxicity
			[57]	Metal Oxide	Oxidative stress

**Table 7:** Endpoints predicted by a dimensionality reduction extracted from the studies gathered.

Reference	NMs Category	Output	Reference	NMs Category	Output
[45]	Polymeric	Arginase: iNOS, cathepsin, IL-10/protein, TNF-α/protein	[107]	Metal Oxide, Quantum Dots	Viability
[52]	Metal, Metal oxide, Quantum Dots	Aggregated	[46]	Metal Oxide	
[124]			[53]		
[39]	Metal, Metal oxide		[51]	Metal, Metal oxide	
[125]			[39]		
[36]	Metal	Cell association	[38]	Metal	Exocytosis
[47]			[113]	Metal Oxide	Membrane integrity
[103]	Carbon-based	Mitotoxicity			

**Table 8:** Endpoints predicted by the ensemble extracted from the studies gathered.

Reference	NMs Category	Output
[65]	Metal, dendrimer, metal oxide, polymeric	Aggregated
[126]	Metal Oxide, Quantum Dots	Aggregated, cellular uptake, viability
[121]	Metal Oxide	Cellular uptake
[102]	Metal, Metal Oxide	MIC, mortality rate, viability
[25]	Metal Oxide	Viability
[84]		
[72]		
[96]	Dendrimers	
[98]	Carbon-based	

**Table 9:** Endpoints predicted by Bayes models extracted from the studies gathered.

Reference	NMs Category	Output
[77]	Metal, Metal oxide, polymeric	Disrupted cellular processes
[59]	Quantum Dots	IC50, viability
[75]	Metal, Metal Oxide	Aggregated
[127]	Carbon-based, Metal, Metal Oxide	
[128]	Metal, Metal Oxide	
[72]	Metal Oxide	Viability
[73]	Metal	
[96]	Dendrimers	

**Table 10:** Studies performing goodness-of-fit, robustness, and predictivity and assessing the applicability domain.

Reference	Algorithm Category	Endpoint Class	Reference	Algorithm Category	Endpoint Class
[35]	Regression	Numerical	[55]	Neural Networks	Numerical
[114]			[126]	Meta	
[116]			[64]	Trees	Binary
[141]			[97]		
[108]			[46]	Regression, Dimen. Red.	Numerical
[112]			[107]		
[110]			[25]	Trees, meta	
[142]			[40]	Neural networks, instance based, trees, regression	
[34]			[28]		Binary
[31]			[102]	Meta, trees, instance based	
[41]			[98]		
[36]	Instance Based	Numerical	[76]	Instance Based	
[62]			[61]		

## Supplementary Materials

The following are available online at https://www.mdpi.com/2079-4991/10/1/116/s1. The equations of model validation and applicability domain are provided in supplementary material.
