# Supplementary material for: Practices and Trends of Machine Learning Application in Nanotoxicology
Source: Nanomaterials (Basel). 2020 Jan 8;10(1):116. doi: 10.3390/nano10010116 (PMC7023261; doi:10.3390/nano10010116)
Supplement: Supplementary file 1 [file nanomaterials-10-00116-s001.pdf]

Supplementary Materials

Practices and Trends of Machine Learning Application in Nanotoxicology

Irini Furxhi [1,2,*], Finbarr Murphy [1,2], Martin Mullins [1,2], Athanasios Arvanitis [3] and Craig A. Poland [4]

1 Department of Accounting and Finance, Kemmy Business School, University of Limerick, Limerick V94PH93, Ireland; finbarr.murphy@transgero.eu (F.M.); martin.mullins@transgero.eu (M.M.)
2 Transgero Limited, Newcastle, Limerick V42V384, Ireland
3 Department of Mechanical Engineering, Environmental Informatics Research Group, Aristotle University of Thessaloniki, Thessaloniki Box 483, 54124, Greece; at.arvanitis@dei.com.gr
4 ELEGI/ Colt Laboratory, Queen's Medical Research Institute, 47 Little France Crescent, University of Edinburgh, Edinburgh EH16 4TJ, Scotland; craig.poland@ed.ac.uk
* Correspondence: irini.furxhi@ul.ie or Irini.furxhi@transgero.eu; Tel.: +353 85 106 9771

1.1 Model validation and applicability domain.

Model validation

Goodness-of-fit

$$R^2 = 1 - \frac{\sum_{i=1}^{n}\left(y_i^{obs} - y_i^{pred}\right)^2}{\sum_{i=1}^{n}(y_i^{obs} - \tilde{y}^{obs})^2} \quad (1)$$

where: y_i^{obs}– observed value for the ith object from training set; y_i^{pred}– predicted value for i[th] object from training set; $\tilde{y}^{obs}$–the mean experimental value of object in the training set

$$R_{adj}{}^2 = 1 - \frac{\sum_{i=1}^{n}\left(y_i^{obs} - y_i^{pred}\right)^2 \,/\, (n-p)}{\sum_{i=1}^{n}(y_i^{obs} - \tilde{y}^{obs})^2 \,/\, (n-1)} \quad (2)$$

where n and p are the total number of samples and the number of parameters in the model, respectively.

Robustness

$$Q_{Loo}^2 = 1 - \frac{\sum_{i=1}^{n}\left(y_i^{obs} - y_i^{predcv}\right)^2}{\sum_{i=1}^{n}(y_i^{obs} - \tilde{y}^{obs})^2} \quad (3)$$

where: y_i^{obs}–observed value for the ith object from training set; y_i^{predcv}– predicted value for i[th] object or the response of the ith object estimated by using a model obtained without using the ith object; $\tilde{y}^{obs}$– the mean experimental value of the object;

$$PRESS = \sum_i (y_i^{obs} - y_i^{predcv})^2 \quad (4)$$

Table 1. Validation performance metrics for classification models. TP = True positive; FP = False positive; TN = True negative; FN = False negative.

$\text{Balanced Accuracy (ACC)} = 0.5 * \left(\frac{TP}{TP+FN} + \frac{TN}{FP+TN}\right) * 100\%$
$\text{Sensitivity (SENS)} = \frac{TP}{TP+FN} * 100\%$
$\text{Specificity (SPEC)} = \frac{TN}{TN+FP} * 100\%$

$$\text{Discriminant Power (DP)} = \frac{\sqrt{3}}{\pi}\left(log\frac{sensitivity}{(1-sensitivity)} + log\frac{specificity}{(1-specificity)}\right)$$

$$Precision = TP/(TP + FP)$$

$$\text{F1 score (F1)} = 2 * \frac{Sensitivity * Precision}{Sensitivity + Precision}$$

$$Matthews\ correlation\ coefficient\ (MCC) = \frac{TP * TN - FP * FN}{\sqrt{(TP + FP)(TP + FN)(TN + FP)(TN + FN)}}$$

Chance testing

$$cR_p^2 = R * \sqrt{R^2 - R_r^2} \tag{5}$$

Predictability

$$MSE = \sum_1^n \frac{(y_i^{obs} - y_i^{pred})^2}{n} \tag{6}$$

where: y_i^{obs}-observed value for the ith object from validation set; y_i^{pred}–predicted value for i[th] object in validation set;and n- total number of samples in training set.

$$Q_{ext}^2 = 1 - \frac{\sum_{i=1}^k \left(y_i^{obs} - y_i^{pred}\right)^2}{\sum_{i=1}^k (y_i^{obs} - \tilde{y}^{obs})^2} \tag{7}$$

where: $\tilde{y}^{obs}$-mean observed value of object in validation set and k-number of samples in validation set.

$$SDEP = \sqrt{\frac{\sum_i (y_i - \hat{y}_{i/i})^2}{n}} \tag{8}$$

where: $\hat{y}_{i/i}$ -response of ith object estimated by using a model obtained withouat using the ith object.

$$RMSEP = \sqrt{\frac{\sum_{i=1}^k \left(y_i^{obs} - y_i^{pred}\right)^2}{k}} \tag{9}$$

$$MAE = \frac{\sum_k \left|y_i^{obs} - y_i^{pred}\right|}{n} \tag{10}$$

$$CCC = \frac{2\sum_{i=1}^k \left(y_i^{obs} - \tilde{y}^{obs}\right)\left(y_i^{pred} - \tilde{y}^{pred}\right)}{\sum_{i=1}^k (y_i^{obs} - \tilde{y}^{obs})^2 + \sum_{i=1}^k \left(y_i^{pred} - \tilde{y}^{pred}\right) + k(\tilde{y}^{obs} - \tilde{y}^{pred})^2} \tag{11}$$

where: $\tilde{y}^{pred}$-mean predicted value of object in validation set.

1.2 Applicability domain (AD)

The leverage is defined as:

$$h_i = x_i^T (X^T X)^{-1} x_i (i = 1,..,n) \tag{12}$$

where h_i is the leverage or hat value of the compound (i) in the descriptor space, x_i is the descriptor raw-vector of the query compound, and X is the descriptor matrix. The superscript T refers to the transpose of the matrix and vector. The observation that a chemical has a leverage value greater than the warning leverage $(h *)$ indicates that the chemical falls outside the applicability domain. The leverage value greater than $h *$ also means that the predicted response is the result of extrapolation of the model and, therefore, may not be reliably set [1,2]. The warning leverage is calculated as follows, where p is the number of model parameters, and n is the number of training data:

$$h *= 3(p + 1)/n \tag{13}$$

The standardized cross-validated residual (ε) is defined as:

$$\varepsilon_i = \frac{\hat{y}_{(LOO)i} - y_i}{S^2} \tag{14}$$

where S^2 is the sample variance of ε_i across all formulations. ε_i characterizes the accuracy of the model estimate of cell association for formulation ' i ' relative to the model estimates for all other formulations. A formulation is considered an outlier if the absolute value of ε_i is greater than 3 [3].

$$APD = \langle d \rangle + Z\sigma \tag{15}$$

where $\langle d \rangle$ is the average Euclidean distance of all distances included in the subset of distances which are lower than the mean value, σ is the standard deviation of all distances included in the subset of distances that are lower than the mean value and Z is an arbitrary empirical cut-off value to control the significance level, usually set to 0.5, which formally places the allowed distance threshold at the mean plus one-half of the standard deviation.

1. Salahinejad, M.; Zolfonoun, E. QSAR studies of the dispersion of SWNTs in different organic solvents. *Journal of Nanoparticle Research* **2013**, *15*, 2028, doi:10.1007/s11051-013-2028-0.
2. Mikolajczyk, A.; Gajewicz, A.; Rasulev, B.; Schaeublin, N.; Maurer-Gardner, E.; Hussain, S.; Leszczynski, J.; Puzyn, T. Zeta Potential for Metal Oxide Nanoparticles: A Predictive Model Developed by a Nano-Quantitative Structure–Property Relationship Approach. *Chemistry of Materials* **2015**, *27*, 2400-2407, doi:10.1021/cm504406a.
3. Walkey, C.D.; Olsen, J.B.; Song, F.; Liu, R.; Guo, H.; Olsen, D.W.H.; Cohen, Y.; Emili, A.; Chan, W.C.W. Protein Corona Fingerprinting Predicts the Cellular Interaction of Gold and Silver Nanoparticles. *ACS Nano* **2014**, *8*, 2439-2455, doi:10.1021/nn406018q.
